# Efficacy of Modified Triple Assessment in Diagnosing Breast Lesions: A Prospective Observational Study

**DOI:** 10.7759/cureus.81538

**Published:** 2025-03-31

**Authors:** Rohan S More, Saurabh Dumbre, Ajit M Dikle

**Affiliations:** 1 Surgery, Government Medical College and Hospital, Dharashiv, IND; 2 General Surgery, Pimpri Chinchwad Municipal Corporation Post Graduate Institute and Yashwantrao Chavan Memorial Hospital, Pune, IND

**Keywords:** breast cancer diagnosis, diagnostic accuracy, fine-needle aspiration cytology, modified triple test (mtt), ultrasound in breast evaluation

## Abstract

Background: Breast lumps are a common clinical presentation, often causing significant anxiety due to the risk of malignancy. Early and accurate differentiation between benign and malignant breast lesions is essential for optimal patient management. The modified triple test (MTT), which replaces mammography with ultrasound in the traditional triple assessment test (TAT), offers a more effective diagnostic approach, particularly in younger women with dense breast tissue. This study evaluates the efficacy of MTT in diagnosing breast lesions.

Methods: A prospective observational study was conducted on 100 female patients aged 15 years and above presenting with palpable breast lumps at South Central Railway Hospital, Secunderabad, India. Patients underwent clinical examination, ultrasound (USG), and fine-needle aspiration cytology (FNAC), with histopathological examination (HPE) as the gold standard. Sensitivity, specificity, positive predictive value (PPV), and negative predictive value (NPV) were calculated for each modality.

Results: The majority of participants were in the 41-50 years age group (38%). Clinical examination demonstrated a sensitivity of 73.08% and specificity of 98.65%. Ultrasound exhibited a sensitivity of 57.69% and specificity of 98.64%. FNAC showed a sensitivity of 84.62% and specificity of 98.65%. MTT demonstrated 100% sensitivity, 98.65% specificity, and 96.30% PPV, significantly outperforming individual modalities.

Conclusion: The MTT is a highly accurate and reliable diagnostic approach for breast lump evaluation, reducing the need for unnecessary biopsies. Its high sensitivity and specificity make it a valuable tool for early breast cancer detection, especially in resource-limited settings.

## Introduction

Breast lumps are among the most common complaints encountered in surgical and gynaecological clinics, often leading to significant anxiety among women due to the potential risk of breast cancer [1]. While a majority of breast lumps are benign, differentiating between benign and malignant lesions is crucial for timely intervention and appropriate management. Breast carcinoma remains a major public health concern, ranking as the most frequently diagnosed cancer and the leading cause of cancer-related mortality in women worldwide. Early and accurate diagnosis plays a pivotal role in improving prognosis and survival outcomes [2].

Clinical examination, imaging modalities, and histopathological evaluation are essential for the diagnostic assessment of breast lumps. Traditionally, the triple assessment test (TAT) has been a cornerstone in breast lump evaluation, comprising clinical examination, mammography, and fine-needle aspiration cytology (FNAC) [3]. However, mammography has limitations, particularly in younger women with dense breast tissue, which can lead to inconclusive results. To overcome these challenges, a modified triple test (MTT) has been proposed, replacing mammography with ultrasound (USG) to improve diagnostic accuracy, especially in premenopausal women [4].

Breast cancer incidence has been steadily rising in India, with an increasing proportion of cases occurring in younger women. Studies indicate an age shift in breast cancer diagnosis, with more cases now being detected in women aged 30-40 years. The aggressive nature of breast carcinoma, coupled with delayed presentation and limited access to healthcare facilities in certain regions, necessitates an efficient, cost-effective, and reliable diagnostic approach [5,6].

The MTT provides a structured, stepwise approach for assessing breast lumps using three primary components: (1) clinical examination (C/E): a detailed history and physical examination help assess lump characteristics such as size, mobility, surface irregularities, and associated skin or nipple changes; (2) ultrasound (USG): a high-resolution imaging technique that differentiates solid vs. cystic lesions and provides additional details about vascularity, margins, and echogenicity; and (3) FNAC or core needle biopsy (CNB): a minimally invasive procedure that provides cytological or histopathological confirmation, aiding in definitive diagnosis.

Studies have demonstrated that MTT has high sensitivity and specificity, making it a reliable alternative to traditional methods. Unlike mammography, ultrasound is more effective in detecting lesions in dense breasts, making it particularly valuable for young women. FNAC, in combination with imaging and clinical findings, further enhances diagnostic accuracy and reduces unnecessary biopsies [7].

The present study was aimed to assess the diagnostic accuracy of the modified triple assessment in the evaluation of breast lumps. By comparing individual components - clinical examination, ultrasound findings, and FNAC results - with final histopathological examination (HPE), this study seeks to establish the reliability of MTT as a first-line diagnostic tool for breast lump evaluation.

## Materials and methods

This prospective observational study was conducted in the Department of General Surgery at South Central Railway Hospital, Lallaguda, Secunderabad, Telangana, India, between 2020 and 2023. The primary objective was to assess the diagnostic accuracy of the MTT in evaluating palpable breast lumps. The study included 100 female patients aged 15 years and above who presented with a clinically detectable breast lump. Patients with a prior history of breast surgery, chemotherapy, or radiotherapy were excluded. Additionally, male patients, those with non-palpable breast lesions detected incidentally on imaging, and pregnant or lactating women were not considered for the study. Ethical clearance was obtained from the Institutional Ethics Committee, and written informed consent was obtained from all participants before enrollment.

Each patient underwent a thorough clinical examination, including detailed history-taking, focusing on age, menstrual and reproductive history, family history, duration of symptoms, and associated complaints. The physical examination assessed lump characteristics, including size, mobility, surface irregularities, and associated skin or nipple changes. The clinical findings were categorized as benign, suspicious, or malignant based on standard clinical criteria. Following the clinical evaluation, all patients underwent high-resolution ultrasonography using a 7-12 MHz linear probe before any invasive procedure. The imaging was performed by a radiologist with expertise in breast imaging, and the findings were categorized using the Breast Imaging-Reporting and Data System (BI-RADS). Lesions classified as BI-RADS 1-3 were considered probably benign, BI-RADS 4 was labelled as indeterminate, and BI-RADS 5-6 were categorized as highly suspicious malignant. The ultrasound examination provided additional details about the lump, including shape, margin, echogenicity, presence of calcifications, and vascularity on Doppler imaging.

FNAC was performed for all patients using a 22-23G needle with a 10-20 mL syringe under aseptic conditions. The aspirated material was spread onto glass slides, air-dried, and stained using Papanicolaou, Giemsa, or hematoxylin & eosin (H&E) stain. The cytological findings were classified using a five-tier system: C1 (inadequate sample), C2 (benign lesion), C3 (atypical, probably benign), C4 (suspicious of malignancy), and C5 (malignant). In cases where FNAC yielded an inconclusive result (C1 category), a core needle biopsy (CNB) was performed using a 14G Tru-Cut needle under ultrasound guidance. The cytology and histopathology slides were reviewed by an experienced pathologist. Patients with suspicious or malignant findings on FNAC/CNB underwent excisional biopsy, lumpectomy, or mastectomy, as clinically indicated, and the final histopathological examination (HPE) report was considered the gold standard for diagnosis.

The data obtained from clinical examination, ultrasound imaging, and FNAC were analyzed to determine the diagnostic accuracy of each individual component of the MTT. Sensitivity, specificity, positive predictive value (PPV), and negative predictive value (NPV) were calculated by comparing MTT findings with final histopathology reports. The overall diagnostic accuracy of MTT was assessed using statistical analysis. The chi-square test was applied to assess the association between different diagnostic modalities and histopathological findings, with a p-value of less than 0.05 considered statistically significant. Statistical analysis was performed using Statistical Product and Service Solutions (SPSS, version 26.0; IBM SPSS Statistics for Windows, Armonk, NY).

The primary outcome measures included the sensitivity, specificity, PPV, and NPV of MTT in detecting malignant breast lumps. Sensitivity was calculated as the proportion of true-positive cases correctly identified, while specificity was the proportion of true-negative cases correctly classified. PPV and NPV were used to assess the likelihood of correctly diagnosing malignant and benign lesions, respectively. The overall diagnostic accuracy was determined by calculating the proportion of correctly classified cases among the total study population.

## Results

A total of 100 patients presenting with palpable breast lumps were evaluated using the MTT, comprising clinical examination, ultrasound imaging, and FNAC/CNB. The study aimed to assess the diagnostic accuracy of each component individually and in combination, comparing findings with the HPE.

The study included 100 participants, with the majority falling in the 41-50 years age group (38%), followed by 31-40 years (35%). A smaller proportion were aged 51-60 years (15%), while 7% were 30 years or younger, and 5% were older than 60 years. In terms of parity, the majority of participants were multiparous (81%), whereas 19% were nulliparous. Regarding menstrual status, 67% of the participants were postmenopausal, while 33% were premenopausal, indicating a higher prevalence of breast lumps among postmenopausal women (Table 1).

**Table 1:** Distribution of study participants according to age, parity, and menstrual status.

Variable		Frequency	Percentage
Age (years)	<=30	7	7.0%
	31-40	35	35.0%
	41-50	38	38.0%
	51-60	15	15.0%
	>60	5	5.0%
Parity	Multiparous	81	81.0%
	Nulliparous	19	19.0%
Menstrual status	Premenopausal	33	33.0%
	Postmenopasual	67	67.0%

The study analyzed the association between parity, menstrual status, and laterality with diagnosis in breast disease using the chi-square test. A statistically significant association (p < 0.01) was found between parity and breast disease diagnosis, with 42.3% of malignant cases occurring in nulliparous women, despite them making up only 19% of the study population. In contrast, only 10.8% of benign cases were in nulliparous women, indicating a higher risk of malignancy in nulliparous individuals. Similarly, menstrual status showed a significant association (p = 0.03), where 50% of malignant cases were in premenopausal women, despite this group comprising only 33% of the total study population. Postmenopausal women had 73% of benign cases, suggesting a lower likelihood of malignancy in this group. Laterality (p = 0.82), however, did not show a significant association with malignancy, as 46.2% of malignant cases and 48.6% of benign cases involved the affected breast, indicating that tumor laterality does not play a crucial role in distinguishing between benign and malignant breast diseases (Table 2).

**Table 2:** Association between diagnosis in breast disease and clinical examination.

Variable		Diagnosis				Total (n=100)		Chi-square	p-value
		Malignant (n=26)		Benign (n=74)					
		n	%	n	%	n	%		
Parity	Multiparous	15	57.7%	66	89.2%	81	81.0%	12.4	< 0.01
	Nulliparous	11	42.3%	8	10.8%	19	19.0%		
Menstrual status	Premenopausal	13	50.0%	20	27.0%	33	33.0%	4.59	0.03
	Postmenopasual	13	50.0%	54	73.0%	67	67.0%		
Laterality	Left	12	46.2%	36	48.6%	48	48.0%	0.0	0.82
	Right	14	53.8%	38	51.4%	52	52.0%		

The histopathological distribution of breast disease cases in the study revealed that fibroadenoma was the most common diagnosis, accounting for 54% of cases, making it the predominant benign breast lesion. This was followed by mammary hamartoma (20%), another benign condition. Among malignant cases, solid papillary carcinoma (8%) and invasive ductal carcinoma (4%) were observed. Less common diagnoses included benign phylloids tumor (3%), borderline phylloids tumor (3%), complex fibroadenoma (2%), and fibroadenoma with cystic changes or adenosis (each 2%). Additionally, infiltrating lobular carcinoma, classical variant (2%), and tubular adenoma (2%) were also noted. The findings suggest that benign breast lesions were far more prevalent than malignant ones, with fibroadenoma being the leading histopathological diagnosis (Table 3).

**Table 3:** Distribution according to histopathological diagnosis.

Cytology	Frequency	Percentage
Fibroadenoma	54	54.0%
Mammary Hamartoma	20	20.0%
Solid Papillary Carcinoma	8	8.0%
Invasive Ductal Carcinoma	4	4.0%
Benign Phylloids Tumor	3	3.0%
Borderline Phylloids Tumor	3	3.0%
Complex Fibroadenoma	2	2.0%
Fibroadenoma with Cystic Changes	2	2.0%
Fibroadenoma with Odenosis	2	2.0%
Infiltrating Lobular Classical Variant	2	2.0%
Tubular Adenoma	2	2.0%

The study compared the effectiveness of different diagnostic modalities in distinguishing between malignant and benign breast lesions, revealing statistically significant associations. Clinical examination detected 73.1% of malignant cases, but missed 26.9%, while correctly identifying 98.6% of benign cases (p < 0.01). Ultrasound had a lower sensitivity, detecting 57.7% of malignant cases, but retained high specificity (98.6% for benign cases) (p < 0.001). FNAC showed better accuracy, identifying 84.6% of malignant cases, with 98.6% specificity (p < 0.001). The MTT, which combines clinical examination, ultrasound, and FNAC, demonstrated 100% sensitivity, correctly identifying all malignant cases while maintaining 98.6% specificity (p < 0.001). The final HPE, considered the gold standard, also confirmed 100% accuracy, making it the definitive diagnostic tool. Overall, the MTT emerged as a highly reliable and accurate diagnostic approach, significantly outperforming individual modalities in detecting malignant breast lesions (Table 4).

**Table 4:** Comparison of diagnostic modalities in differentiating malignant and benign breast lesions.

Variable		Diagnosis				Total (n=100)		Chi-square	p-value
		Malignant (n=26)		Benign (n=74)					
		n	%	n	%	n	%		
Clinical Examination	Malignant (+)	19	73.1%	1	1.4%	20	20.0%	61.86	<0.01
	Benign (-)	7	26.9%	73	98.6%	80	80.0%		
Ultrasound Result	Malignant (+)	15	57.7%	1	1.4%	16	16.0%	50.22	<0.001
	Benign (-)	11	42.3%	73	98.6%	84	84.0%		
FNAC Result	Malignant (+)	22	84.6%	1	1.4%	23	23.0%	75.31	<0.001
	Benign (-)	4	15.4%	73	98.6%	77	77.0%		
Modified Triple test	Malignant (+)	26	100.0%	1	1.4%	27	27.0%	94.99	<0.001
	Benign (-)	0	0.0%	73	98.6%	73	73.0%		
Final HPE	Malignant (+)	26	100.0%	0	0.0%	26	26.0%	100.0	<0.001
	Benign (-)	0	0.0%	74	100.0%	74	74.0%		

The diagnostic accuracy of various modalities in detecting malignant breast lesions was assessed using sensitivity, specificity, PPV, and NPV. Among individual modalities, clinical examination had a sensitivity of 73.08%, meaning it correctly identified 73.08% of malignant cases, while its specificity was high (98.65%), ensuring a low false-positive rate. Ultrasound showed lower sensitivity (57.69%) but retained high specificity (98.64%), indicating its role as a complementary diagnostic tool rather than a primary screening method. FNAC performed better, with a sensitivity of 84.62% and a PPV of 95.62%, confirming its effectiveness in diagnosing malignancy. The MTT, which integrates clinical examination, ultrasound, and FNAC, exhibited 100% sensitivity and 98.65% specificity, making it a highly reliable diagnostic tool. As expected, the final HPE was the gold standard, achieving 100% sensitivity, specificity, PPV, and NPV. These findings emphasize that, while clinical examination, ultrasound, and FNAC have diagnostic value, the MTT significantly enhances accuracy and reliability, approaching near perfect diagnostic performance before final histopathological confirmation (Table 5).

**Table 5:** Diagnostic accuracy of various modalities in detecting malignant breast lesions. FNAC: fine-needle aspiration cytology; HPE: histopathological examination; NPV: negative predictive value; PPV: positive predictive value

Variable	Sensitivity	Specificity	PPV	NPV
Clinical Examination	73.08%	98.65%	95.0%	91.25%
Ultrasound Result	57.69%	98.64%	93.75%	86.90%
FNAC Result	84.62%	98.65%	95.62%	94.81%
Modified Triple Test	100%	98.65%	96.30%	100%
Final HPE	100%	100%	100%	100%

The distribution of tumor size in benign and malignant breast lesions shows that tumors measuring 4 × 3 cm (34.6%) and 6 × 5 cm (23.1%) were more frequently malignant, while benign tumors were predominantly 5 × 5 cm (17.6%) and 5 × 6 cm (14.9%) (Table 6).

**Table 6:** Distribution of tumor size as measured by USG in benign and malignant breast lesions.

Tumor size (cm) as measured by USG	Benign		Malignant		Total	
	n	%	n	%	n	%
3*3	2	2.7%	0	0.0%	2	2.0%
4*2	1	1.4%	0	0.0%	1	1.0%
4*3	4	5.4%	9	34.6%	13	13.0%
4*4	0	0.0%	2	7.7%	2	2.0%
5*3	0	0.0%	2	7.7%	2	2.0%
5*4	7	9.5%	2	7.7%	9	9.0%
5*5	13	17.6%	0	0.0%	13	13.0%
5*6	11	14.9%	0	0.0%	11	11.0%
6*4	4	5.4%	3	11.5%	7	7.0%
6*5	10	13.5%	6	23.1%	16	16.0%
6*6	1	1.4%	0	0.0%	1	1.0%
6*7	1	1.4%	0	0.0%	1	1.0%
7*4	3	4.1%	0	0.0%	3	3.0%
7*5	4	5.4%	0	0.0%	4	4.0%
7*6	4	5.4%	1	3.8%	4	4.0%
7*7	2	2.7%	0	0.0%	2	2.0%
8*5	1	1.4%	0	0.0%	1	1.0%
8*6	6	8.1%	1	3.8%	7	7.0%
Total	74	100.0%	26	100.0%	100	100.0%

## Discussion

Breast cancer is the most common malignancy in females and a leading cause of cancer-related deaths worldwide. Early detection plays a crucial role in improving survival rates, and mammography remains a cornerstone in breast cancer assessment [8,9]. It is effective in characterizing breast masses and detecting occult lesions, with a reported sensitivity of approximately 90% and specificity of 88%. However, its false-negative rate of 8-10% highlights the need for adjunctive diagnostic methods. The MTT, which combines clinical examination, ultrasound, and FNAC, has emerged as a more reliable approach, reducing the likelihood of missed malignancies [10].

In the present study, the majority of patients (38%) belonged to the 41-50 years age group, followed by 35% in the 31-40 years range. This aligns with studies by Karim et al. [11] and Solanki et al. [12], who found that breast cancer is most prevalent in women between 36 and 45 years. Younger patients tend to present with benign conditions, whereas malignancies are more frequently diagnosed in older individuals. These findings reinforce the importance of age-targeted screening programs to improve early detection rates. Parity has been identified as a significant factor in breast cancer risk. In this study, among 19 nulliparous women, 11 (42.3%) were diagnosed with carcinoma, suggesting an increased risk in nulliparous individuals. Conversely, malignancies were also prevalent among multiparous women, demonstrating a complex relationship between reproductive factors and breast cancer risk. These findings corroborate existing literature, which suggests that both early and late pregnancies may influence hormonal exposure and subsequent cancer development [13].

The most common benign tumor observed was fibroadenoma, consistent with previous studies where it constituted a significant proportion of benign breast lesions. Studies by Solanki et al. [12] and Lingaraju et al. [14] have reported similar findings, with fibroadenomas comprising over 50% of benign breast cases. In contrast, solid papillary carcinoma and invasive ductal carcinoma were the most frequent malignant histopathological findings, reaffirming the importance of comprehensive diagnostic evaluation. Clinical examination in this study had a sensitivity of 73.08%, indicating that 26.92% of malignancies were missed. Despite this limitation, its specificity (98.65%) and high NPV highlight its reliability as an initial screening tool. These findings align with those of Kaufman et al. [15] and Ahmad et al. [16], who reported similar sensitivity and specificity for triple assessment.

Ultrasound demonstrated 57.69% sensitivity in detecting malignancies, reinforcing its role as an adjunct rather than a standalone diagnostic tool. Despite its high specificity (98.64%), the sensitivity remains operator-dependent. Advances in elastography and contrast-enhanced ultrasound have been shown to improve diagnostic precision, as noted in studies by Vaithianathan et al. [10] and de Boniface et al. [17]. Future studies should explore the integration of these technologies into routine screening protocols. FNAC exhibited 84.62% sensitivity and 98.65% specificity, with a PPV of 95.62%, confirming its high diagnostic value. This aligns with studies by Karim et al. [11], Khemka et al. [18], and Vaithianathan et al. [10], who found FNAC to be a highly accurate diagnostic tool. However, FNAC is operator-dependent, and inconclusive or false-negative results necessitate histopathological confirmation.

The MTT demonstrated 100% sensitivity, 98.65% specificity, and a PPV of 96.30%, making it a highly effective diagnostic strategy. Studies by Solanki et al. [12] and Kaufman et al. [15] have similarly emphasized its reliability, reducing the need for unnecessary biopsies while ensuring accurate malignancy detection. The MTT's performance underscores its utility in preoperative decision-making and improving patient outcomes. Histopathological examination remains the definitive diagnostic method, confirming all malignant cases with 100% sensitivity and specificity. Studies by Nigam et al. [19] and Solanki et al. [12] have corroborated these findings, emphasizing the necessity of HPE for final diagnosis. Additionally, tumor size was found to have a significant correlation with malignancy, whereas laterality did not show a strong association, consistent with the results of Yang et al. [20] and Shetty et al. [21].

The study is limited by its small sample size and single-center design, which may affect the generalizability of findings. Additionally, the accuracy of USG and FNAC is operator-dependent, potentially introducing variability in results. Future multicenter studies with larger cohorts are needed for broader validation.

## Conclusions

The present study indicates that the MTT may offer a modest advantage over individual diagnostic modalities in detecting breast malignancies. While clinical examination and ultrasound provide valuable initial insights, FNAC and histopathological confirmation remain crucial for definitive diagnosis. The findings reinforce the importance of a multimodal approach in breast cancer detection and suggest that advances in imaging technology could further enhance diagnostic accuracy. Future research should focus on refining risk stratification protocols and incorporating newer imaging techniques to improve early detection and patient outcomes.
